# Mutations at opposite ends of the DIII/S4-S5 linker of sodium channel Na_V_1.7 produce distinct pain disorders

**DOI:** 10.1186/1744-8069-6-24

**Published:** 2010-04-29

**Authors:** Xiaoyang Cheng, Sulayman D Dib-Hajj, Lynda Tyrrell, Dowain A Wright, Tanya Z Fischer, Stephen G Waxman

**Affiliations:** 1 Department of Neurology and Center for Neuroscience & Regeneration Research, Yale University School of Medicine, New Haven, CT, USA; 2 Rehabilitation Research Center, Veterans Affairs Connecticut Healthcare System, West Haven, CT, USA; 3 Division of Rheumatology & Immunology, Children's Hospital of Central California, Madera, CA, USA

## Abstract

**Background:**

Two groups of gain-of-function mutations in sodium channel Na_V_1.7, which are expressed in dorsal root ganglion (DRG) neurons, produce two clinically-distinct pain syndromes - inherited erythromelalgia (IEM) and paroxysmal extreme pain disorder (PEPD). IEM is characterized by intermittent burning pain and skin redness in the feet or hands, triggered by warmth or mild exercise, while PEPD is characterized by episodes of rectal, ocular and mandibular pain accompanied with skin flushing, triggered by bowel movement and perianal stimulation. Most of the IEM mutations are located within channel domains I and II, while most of the PEPD mutations are located within domains III and IV. The structural dichotomy parallels the biophysical effects of the two types of mutations, with IEM mutations shifting voltage-dependence of Na_V_1.7 activation in a hyperpolarized direction, and PEPD mutations shifting fast-inactivation of Na_V_1.7 in a depolarized direction. While four IEM and four PEPD mutations are located within cytoplasmic linkers joining segments 4 and 5 (S4-S5 linkers) in the different domains (IEM: domains I and II; PEPD: domains III and IV), no S4-S5 linker has been reported to house both IEM and PEPD mutations thus far.

**Results:**

We have identified a new IEM mutation P1308L within the C-terminus of the DIII/S4-S5 linker of Na_V_1.7, ten amino acids from a known PEPD mutation V1298F which is located within the N-terminus of this linker. We used voltage-clamp to compare the biophysical properties of the two mutant channels and current-clamp to study their effects on DRG neuron excitability. We confirm that P1308L and V1298F behave as prototypical IEM and PEPD mutations, respectively. We also show that DRG neurons expressing either P1308L or V1298F become hyperexcitable, compared to DRG neurons expressing wild-type channels.

**Conclusions:**

Our results provide evidence for differential roles of the DIII/S4-S5 linker N- and C-termini in channel inactivation and activation, and demonstrate the cellular basis for pain in patients carrying these mutations.

## Background

Gain-of-function mutations of voltage-gated sodium channel Na_V_1.7 have been linked to two familial pain disorders: inherited erythromelalgia (IEM) and paroxysmal extreme pain disorder (PEPD) [1,2], which have distinct clinical characteristics. IEM is characterized by intermittent burning pain and skin redness in the distal extremities, triggered by warmth or mild exercise [1,3]. In contrast, PEPD is characterized by episodes of rectal, ocular and mandibular pain accompanied by skin flushing, triggered by bowel movement and perianal stimulation [4]. IEM is usually unresponsive to pharmacotherapy, while PEPD pain is often relieved by carbamazepine [2,4-6].

Na_V_1.7 is preferentially expressed in dorsal root ganglion (DRG) neurons and sympathetic ganglion neurons, for example superior cervical ganglia [7-9], and produces tetrodotoxin (TTX)-sensitive and fast-inactivating inward currents [8,10]. Na_V_1.7 channels respond to small, slow depolarizations by producing ramp currents which could boost weak stimuli to reach threshold for action potentials [11]. Thus, Na_V_1.7 channels contribute to setting action potential threshold in DRG neurons [12].

All but one of the known IEM mutations are localized to domains I and II of the Nav1.7 channel, while all but one of the known PEPD mutations are localized to domains III and IV [13]. All IEM mutations studied thus far produce a hyperpolarizing shift of activation of Na_V_1.7 [14-24]. In contrast, all PEPD mutations studied thus far impair channel fast-inactivation [2,5,6,25]. Intriguingly, the clustered distribution of the IEM and PEPD mutants along the channel's polypeptide parallels the effects on channel gating. Both IEM and PEPD mutations increase excitability of DRG neurons [5,6,18,24,26], providing a cellular basis for pain symptoms in patients with these pain disorders.

Here, we report a new IEM mutation in Na_V_1.7 (P1308L) within the C-terminus of the S4-S5 linker of domain III (DIII/S4-S5). P1308L is 9-10 amino acids distal to three PEPD mutations (V1298F, V1299D, and V1299F), which are located within the N-terminus of DIII/S4-S5. To better understand how these mutations within the same part of the channel cause distinct pain symptoms, we compared electrophysiological properties of P1308L (IEM) and V1298F (PEPD) mutant channels using voltage- and current-clamp recordings. We show that these two mutations, both within the DIII/S4-S5 linker of the channel, have differential effects on activation and inactivation, and demonstrate that both increase the excitability of DRG neurons.

## Results

### Clinical phenotype and identification of the P1308L mutation in Exon 21

**Figure 1:**
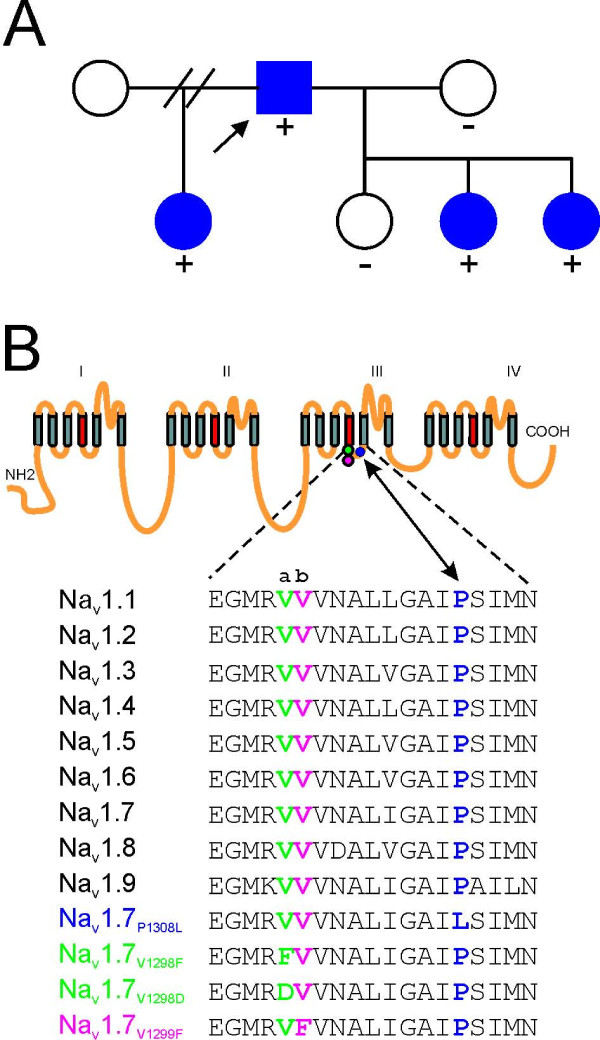
**Identification of the P1308L substitution in Na_V_1.7 in a family with IEM**. **A, **The family pedigree of the proband carrying P1308L mutation. Circles denote females; squares denote males. The proband is indicated by an arrow. Blue symbols indicate subjects affected with IEM. A (+) symbol denotes subjects heterozygous for the P1308L mutation in exon 21 and a (-) symbol denotes subjects without the mutation. **B, **Schematic of the topology of sodium channel and sequence alignment of DIII/S4-S5 linker of human sodium channels. The location of P1308L is indicated with blue symbol (filled blue circle), and sites of V1298F and V1299F are indicated with green and magenta symbols (filled green circle and filled pink circle), respectively. Sequence alignment revealed that both P1308 and V1298 are highly conserved among human voltage-gated sodium channels.

The proband is a Hispanic male of Puerto Rican origin who presented with a history of episodes of burning pain in both feet, beginning around the age of 2 years. The patient reported that warmth triggers pain and that cooling provides relief, and prefers wearing open toed shoes without socks, even during the winter months. Pharmacotherapy has not relieved these symptoms. Three of the proband's children display similar symptoms of pain in the feet, evoked by warmth, with early age of onset (Figure 1A). Similar to the proband, the children's pain is ameliorated by cooling their feet, and they do not tolerate wearing socks or shoes.

Sequence analysis of *SCN9A *amplicons identified a nucleotide change ca.3971C>T in Exon 21, which leads to a substitution of proline 1308 of the reference Na_V_1.7 sequence [8] by leucine (P1308L). This mutation segregates with the affected members in this family but not with unaffected family members (Figure 1A) and was not present in 100 control alleles. P1308 is located within the C-terminus of the S4-S5 linker of domain III in Na_V_1.7 (DIII/S4-S5), and is highly conserved among all human sodium channels (Figure 1B).

Three PEPD mutations (V1298F, V1298D, and V1299F) have been reported within the N-terminus of the same DIII/S4-S5 linker, 9-10 amino acids upstream from P1308 [2]. V1298 is also highly conserved among all human sodium channels (Figure 1B).

### Voltage-clamp electrophysiology

**Figure 2:**
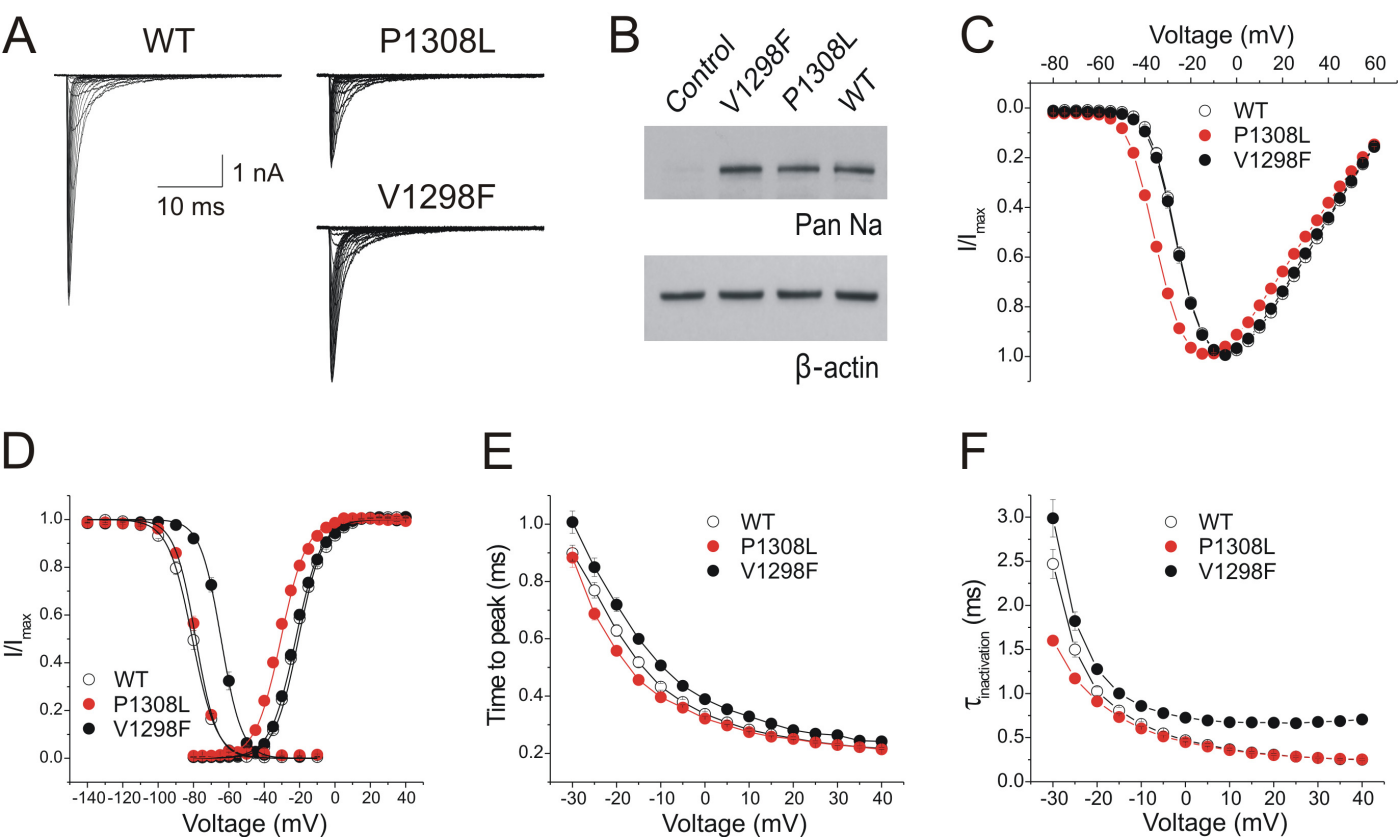
**P1308L and V1298F exhibit different effects on voltage-dependence of activation and fast inactivation**. **A**, Representative families of traces of Na^+ ^currents (I_Na_) from voltage-clamped HEK293 cells stably expressing wild-type (WT), P1308L, or V1298F Na_V_1.7_R _channels. Cells were held at -100 mV, and Na^+ ^currents were elicited by step depolarizations from -80 to +60 mV in 5 mV increments every 5 seconds. **B**. Western blot analysis of Na_V_1.7_R _WT and mutant channels in transfected HEK 293 cells. The loading variation was eliminated by normalizing the intensities of sodium channels with the intensities of β-actin of corresponding lanes. No statistic difference was observed between WT and mutant channels. **C**, Normalized peak current-voltage relationship for WT (n = 29), P1308L (n = 25), and V1298F (n = 26) Na_V_1.7_R _channels. **D**, Comparison of the voltage-dependent activation and steady-state fast inactivation of WT, P1308L, and V1298F channels. A hyperpolarizing shift (-9.6 mV) of activation was observed in the P1308L mutant channel, while the V1298F mutant channel showed a depolarizing shift (+16.1 mV) of steady-state fast-inactivation. **E, **Activation kinetics (measured as time-to-peak) of P1308L (n = 25) were faster at -20 mV and -15 mV, compared to WT channels (n = 29), whereas V1298F channels (n = 26) showed slower activation kinetics from -20 to +40 mV. **F, **Open-state fast-inactivation kinetics were measured by single-exponential fitting of the decay phases of I_Na _as shown in (**A**). When compared with wild-type channels (n = 25), V1298F mutant channels (n = 26) significantly slowed the inactivation kinetics from -25 to +40 mV, whereas P1308L mutation (n = 19) showed faster inactivation kinetics at -30 and -25 mV.

The electrophysiological properties of wild-type (WT) or mutant Na_V_1.7_R _channels were investigated using whole-cell voltage-clamp recording in HEK293 cells stably expressing WT, P1308L (IEM), or V1298F (PEPD) channels. Figure 2A shows representative inward Na^+ ^currents recorded from cells stably expressing WT, P1308L, or V1298F Na_V_1.7_R _channels. Pilot experiments showed that cells expressing mutant channels produced smaller currents than cells expressing WT channels. To determine whether the reduced P1308L current density is the result of intrinsic effects of the mutations or the effect of the site of integration of the channel within the HEK 293 genome, we measured the sodium current density in transiently transfected HEK 293 cells. V1298F has previously been reported [25] to produce smaller currents than WT channels in transiently transfected HEK 293 cells, and we did not follow up on it in this study. The current density of P1308L in transiently-transfected HEK 293 cells is significantly smaller than that of WT channels (WT: 285 ± 46 pA/pF, n = 9; P1308L: 90 ± 14, n = 11, *p *= 0.003, two-tailed student's t test). To examine whether the smaller current densities of mutant channels P1308L and V1298F were due to lower protein expression, we used Western blot to assay channel protein levels in transiently transfected HEK 293 cells. When compared with WT channels (set as 100%), the protein levels of mutant channels were 85 ± 12% (n = 3, *p *= 0.526) for P1308L and 123 ± 15% (n = 2, *p *= 0.352) for V1298F channels (Figure 2B), suggesting that the smaller currents from mutant channels are not caused by reduced channel synthesis in HEK 293 cells.

To minimize the difference of voltage error between WT and mutant P1308L currents, HEK 293 cells producing currents larger than 10 nA (i.e. representing ≥ 4X the mean peak currents of P1308L channels) were excluded from the analysis; none of the cells expressing P1308L or V1298F channels produced sodium currents larger than 10 nA. Despite the exclusion of cells producing large WT currents, the current densities of mutant channels were still significantly smaller than that of WT channels (WT: 351 ± 25 pA/pF, n = 29; P1308L: 146 ± 15 pA/pF, n = 25, *p *< 0.001 vs WT; V1298F: 183 ± 16 pA/pF, n = 26, *p *< 0.001 vs WT; non-parametric Kruskal-Wallis statistical test).

Like all IEM mutant channels characterized thus far, P1308L mutation caused a hyperpolarizing shift (-9.6 mV) of activation (WT: V_1/2,act _= -21.8 ± 0.7 mV, *k *= 6.95 ± 0.11, n = 29; P1308L: V_1/2,act _= -31.4 ± 0.5 mV, *k *= 7.33 ± 0.09, n = 25; p < 0.001 for V_1/2,act _and p = 0.012 for *k*) whereas V1298F, the PEPD mutation, had no effect on activation (V_1/2,act _= -22.5 ± 0.7 mV, *p *= 0.680, and *k *= 6.83 ± 0.07, *p *= 0.754, n = 26) (Figure 2C and 2D, Table 1). P1308L mutation did not affect the midpoint (V_1/2,fast_) of steady-state fast-inactivation, but altered the slope of fast-inactivation curve (WT: V_1/2,fast _= -80.6 ± 1.1 mV, *k *= 6.32 ± 0.14, n = 13; P1308L: V_1/2,fast _= -78.9 ± 0.7 mV, *p *= 0.422, *k *= 5.65 ± 0.11, *p *= 0.002) (Figure 2D, Table 1). Like other PEPD mutations characterized to date, V1298F channels showed a depolarizing shift (+16.1 mV) of steady-state fast-inactivation and a steeper inactivation curve (V_1/2,fast _= -64.5 ± 0.9 mV, *p *< 0.001, *k *= 5.45 ± 0.15, *p *< 0.001, n = 13) (Figure 2D, Table 1).

**Table 1:** Parameters of voltage-dependent activation and steady-state fast-inactivation of WT, P1308L, and V1298F Na_V _1.7_R _channels.

	Activation			Steady-state fast inactivation		
		
	V_1/2, act_	*k*	n	V_1/2, fast_	*k*	n
WT	-21.8 ± 0.7	6.95 ± 0.11	29	-80.6 ± 1.1	6.32 ± 0.13	14
P1308L	-31.4 ± 0.5*	7.33 ± 0.09*	25	-78.9 ± 0.7	5.65 ± 0.11*	13
V1298F	-22.5 ± 0.7	6.83 ± 0.07	26	-64.5 ± 0.9*	5.45 ± 0.15*	13

* *p *< 0.05 P1308L or V1298F channels vs WT Na_V_1.7_R _channels.

Activation kinetics (measured as time-to-peak) of P1308L channels were faster at -20 and -15 mV, whereas V1298F mutant channels were slower (between -20 to +40 mV) compared to WT channels (Figure 2E). The kinetics of open-state fast-inactivation were analyzed by mono-exponential fit of the decaying phase of Na^+ ^currents in Figure 2A. V1298F mutant channels exhibited slower inactivation kinetics, compared to WT, from -25 to +40 mV, whereas P1308L channels showed faster kinetics at -30 and -25 mV (Figure 2F). The faster inactivation kinetics of P1308L may be due to enhanced activation of P1308L channels, since open-state fast-inactivation is coupled to the activation state of channels [27].

**Figure 3:**
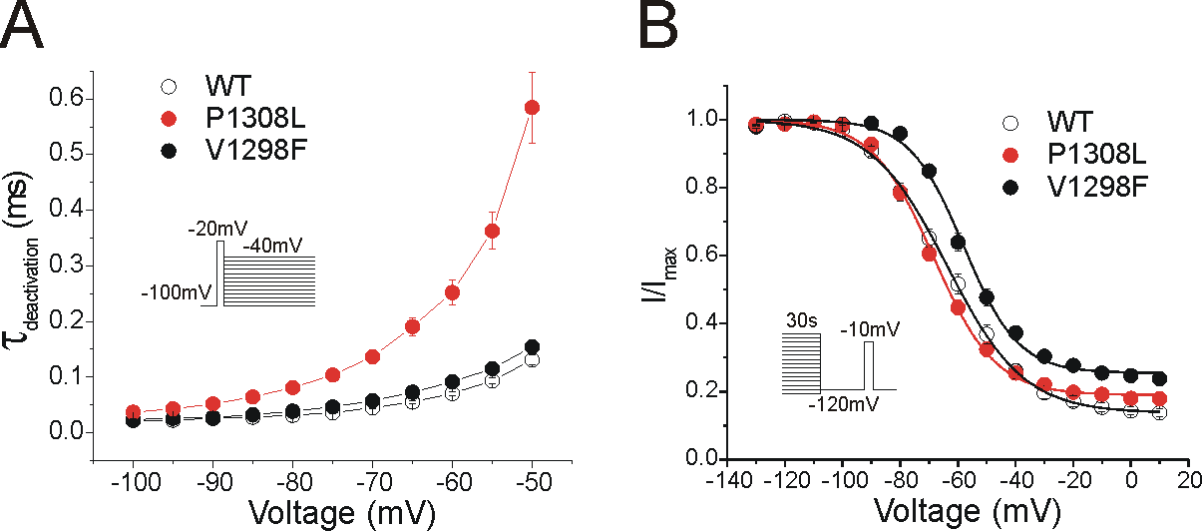
**The P1308L and V1298F mutations have different effects on channel deactivation and slow-inactivation**. **A**, To measure deactivation kinetics, cells were held at -100 mV and tail currents were generated by a brief 0.5-ms depolarization to -20 mV followed by a series of repolarizations ranging from -100 to -40 mV to elicit tail currents. P1308L mutant channels (n = 11) showed slower deactivation kinetics than WT channels (n = 15) at all tested potentials, whereas V1298F had no effect on deactivation kinetics (n = 15). **B, **Steady-state slow-inactivation was examined by a series of prepulses (30 s) from -130 to +10 mV followed by 100-ms return pulse to -120 mV, then a 20-ms test pulse to -10 mV. The V1298F mutation shifted the slow-inactivation curve to more positive potential, whereas the P1308L mutation had no effect on V_1/2,slow_, slow. Both mutations increased the fraction of channels resistant to slow-inactivation (R_resist_).

Deactivation kinetics reflect the transition of channels from the open state to the closed state. As with most IEM mutations, P1308L mutant channels showed slow deactivation of Na^+ ^currents at all potentials tested, indicating that the mutant channel resides longer in the open state, whereas V1298F mutation had no effect on deactivation kinetics (Figure 3A).

Steady-state slow-inactivation develops over a long time frame (from seconds to minutes) upon sustained stimulation. The slow-inactivation of sodium channels was evaluated using 30-s prepulses at potentials ranging from -130 to +10 mV. P1308L did not significantly affect the voltage-dependence of slow-inactivation of mutant channels (WT: V_1/2,slow _= -63.8 ± 1.7 mV, n = 14; P1308L: V_1/2,slow _= -68.4 ± 1.2 mV, n = 10; *p *= 0.072, Table 2, Figure 3B), while V1298F depolarized the slow-inactivation curve of mutant channels by +6 mV (V1298F: V_1/2,slow _= -57.8 ± 1.1 mV, n = 12; *p *= 0.011, Table 2, Figure 3B). Both mutant channels decreased the slope factor of the inactivation curve, and increased the fraction of channels resistant to slow inactivation (R_resist_, expressed as % of maximal current and calculated as offset (A) × 100%. For WT: *k *= 12.6 ± 0.3, R_resist _= 14.0 ± 1.2%, n = 14; P1308L: *k *= 10.5 ± 0.3, *p *< 0.001, R_resist _= 19.1 ± 1.4%, n = 10, *p *= 0.03; V1298F: *k *= 9.2 ± 0.3, *p *< 0.001, R_resist _= 25.6 ± 1.5%, n = 12, *p *< 0.001) (Table 2, Figure 3B).

**Table 2:** Parameters of steady-state slow-inactivation of WT, P1308L, and V1298F Na_V_1.7_R _channels.

	Steady-state slow inactivation			
	
	V_1/2, slow_	*k*	R_resist_(%)	n
WT	-63.8 ± 1.7	12.6 ± 0.3	14.0 ± 1.2	14
P1308L	-68.4 ± 1.2	10.5 ± 0.3*	19.1 ± 1.4*	10
V1298F	-57.8 ± 1.1*	9.2 ± 0.3*	25.6 ± 1.5*	12

* *p *< 0.05 P1308L or V1298F channels vs WT Na_V_1.7_R _channels.

**Figure 4:**
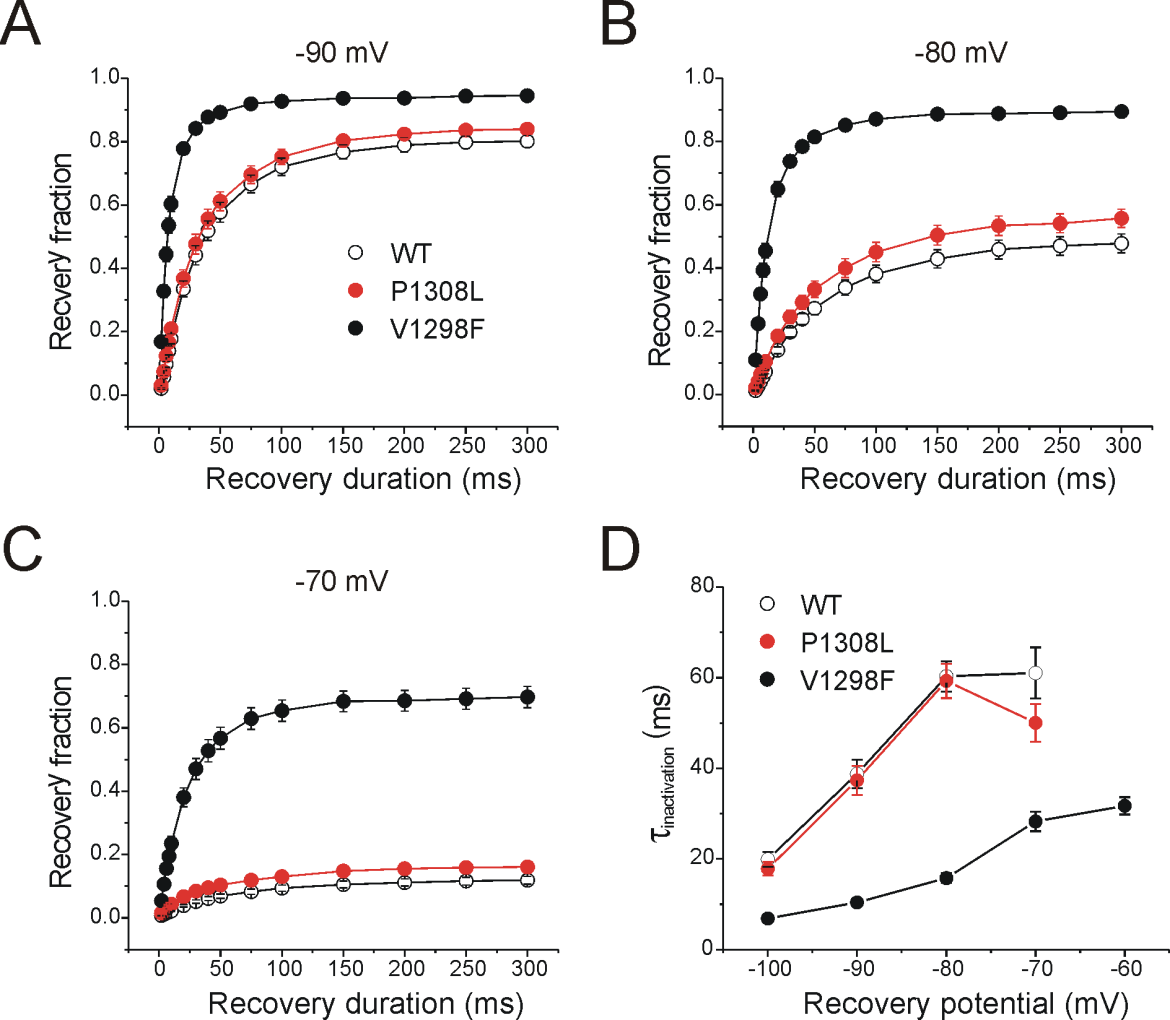
**The P1308L and V1298F mutations have different effects on repriming**. Cells were held at -100 mV, and fast-inactivation was initiated by a 20-ms depolarization to 0 mV, followed by a recovery period (2-300 ms) at a recovery potential. The available channels were then measured with a 10-ms test pulse at 0 mV. Recovery fraction was calculated by normalizing the peak currents in response to test pulses, to peak currents of prepulses after various recovery durations (2-300 ms) at different recovery potentials, and plotted as a function of recovery potentials. **A, B, and C, **V1298F mutant channels show higher recovery fractions than WT channels at recovery potentials from -90 to -70 mV, whereas P1308L channels do not affect recovery fraction. **D, **Repriming kinetics was calculated by mono-exponential fits of the recovery rate at different recovery duration. V1298F channels showed faster repriming kinetics than WT channels at all testing potentials, whereas P1308L had no effect on the repriming kinetics.

Repriming kinetics reflect the recovery rate of channels from fast-inactivation state, and in the case of Na_V_1.7, repriming kinetics may regulate how fast a neuron can repetitively fire [28]. The P1308L mutation had no significant effect on recovery fractions or on repriming kinetics at tested potentials (Figure 4A, B, C, and 4D). In contrast, V1298F mutant channels showed faster repriming kinetics at all tested potentials (Figure 4D) and higher recovery fraction at recovery potentials from -90 to -60 mV (Figure 4A, B and 4C). The larger recovered fraction of V1298F channels is related to the depolarizing shift of fast-inactivation, which increases the proportion of channels available for activation at these recovery potentials. Faster repriming kinetics and larger recovery fraction of V1298F channels are expected to endow neurons housing this mutation with the ability to fire at higher frequency compared to wild type channels.

**Figure 5:**
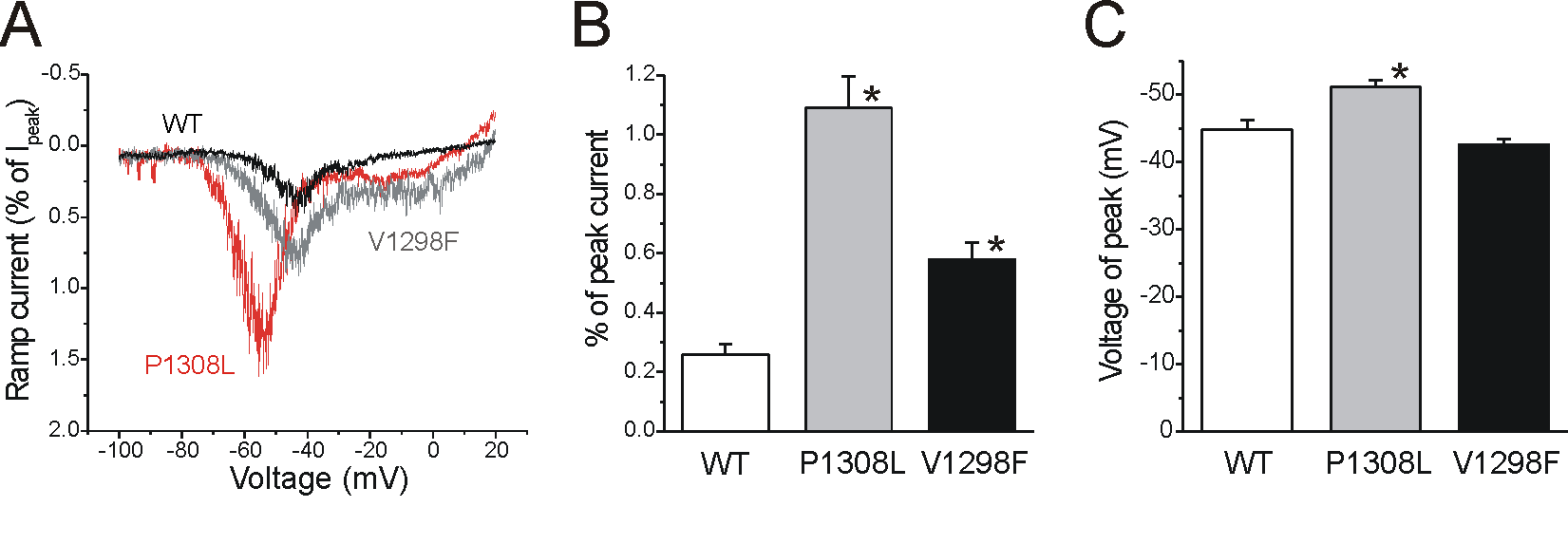
**The P1308L and V1298F mutations enhance the response to slow ramp depolarization**. HEK293 cells were held at -100 mV and a depolarizing voltage ramp from -100 mV to +20 mV was applied at a rate of 0.2 mV/ms. **A, **Representative ramp currents from WT (black), P1308L (red), and V1208F (grey) channels. Currents were normalized to maximal peak currents elicited by step depolarizations in Figure 1B. **B, **Both P1308L and V1298F mutations significantly increase the relative amplitude of ramp currents (WT: 0.26 ± 0.03%, n = 12; P1308L: 1.09 ± 0.11%, n = 15, *p *< 0.001 vs WT; V1298F: 0.58 ± 0.06%, n = 16, *p *= 0.015 vs WT). **C, **The potential of peak ramp currents was more negative in P1308L mutant channels than in WT and V1298F channels (WT: -44.8 ± 1.4 mV, n = 12; P1308L: -51.1 ± 0.9 mV, n = 15, *p *< 0.001 vs WT; V1298F: -42.5 ± 0.8 mV, n = 16, *p *= 0.280 vs WT).

Na_V_1.7 channels activate in response to small, slow depolarization, which allows them to amplify weak stimuli, e.g. generator potentials, bringing the membrane potential closer to the threshold for initiation of action potentials [11]. Therefore, we examined the effects of mutations on the channel response to slow ramp depolarization (600 ms ramp depolarization from -100 mV to +20 mV; 0.2 mV/ms). Figure 5A shows representative ramp currents recorded from cells expressing WT, P1308L and V1298F channels. The ramp currents, measured as percentage of peak current, generated by P1308L channels were about 4X larger than those of WT channels (WT: I_ramp _= 0.26 ± 0.03%, n = 12; P1308L: I_ramp _= 1.09 ± 0.11%, n = 15, p < 0.001) (Figure 5B). Consistent with the hyperpolarized shift of voltage-dependence of activation, P1308L mutation also shifted the peak of the ramp currents to more negative potentials (WT: V_ramp _= -44.8 ± 1.4 mV, n = 12; P1308L: V_ramp _= -51.1 ± 0.9 mV, n = 15, p < 0.001) (Figure 5C). Compared to WT channels, V1298F channels also produced 2X larger ramp currents, but had no effect on the voltage for peak ramp currents (V1298F: I_ramp _= 0.58 ± 0.06%, n = 16, *p *= 0.015; V_ramp _= -42.5 ± 0.8 mV, n = 16, *p *= 0.280) (Figure 5B, C).

### Current-clamp electrophysiology

**Figure 6:**
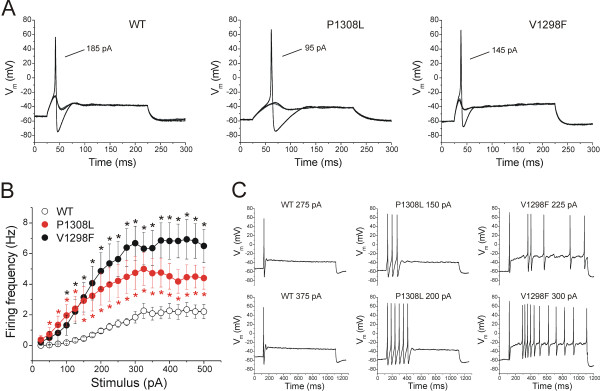
**Excitability is increased in DRG neurons transfected with the P1308L or V1298F mutant channels**. **A, **Responses of DRG neurons expressing WT, P1308L, or V1298F channels to a series of current stimuli with 5-pA increment. DRG neurons expressing P1308L channels showed a lower action potential threshold (P1308L: 122 ± 10 pA, n = 50, *p *< 0.001 vs WT: 188 ± 14 pA, n = 38), while expression of V1298F did not significantly change the action potential threshold (V1298F: 154 ± 18 pA, n = 27, *p *= 0.215 vs WT). **B, **The mean firing frequency of DRG neurons expressing WT, P1308L, or V1298F channels in response to a series of 1-s current injections ranging from 25 to 500 pA with 25-pA increments. Both P1308L and V1298F elevated firing frequency of transfected DRG neurons. * indicates *p *< 0.05 for P1308L vs WT channels, * indicates *p *< 0.05 for V1298F vs WT channels. **C, **Representative traces showing responses to current stimuli approximately 1.5× (top) and 2× (bottom) action potential threshold, recorded from DRG neurons expressing WT, P1308L, or V1298F channels from (**A**).

To examine the effects of the P1308L and V1298F mutations on DRG neuron excitability, current-clamp recordings were performed on neonatal rat DRG neurons transfected with WT, P1308L, or V1298F constructs combined with GFP. The input resistance (R_input_), resting membrane potential (RMP), current threshold of action potential, and firing frequency in small DRG neurons were examined. Expression of mutant channels did not change R_input _and RMP of DRG neurons (Table 3). However, expression of P1308L channels decreased the current threshold of action potential in DRG neurons (WT: 188 ± 14 pA, n = 38; P1308L: 122 ± 10 pA, n = 50, *p *< 0.001, Table 3, Figure 6A), whereas the action potential threshold of DRG neurons expressing V1298F channels was not significantly different from that of neurons expressing WT channels (V1298F: 154 ± 18, n = 27, *p *= 0.215, Table 3, Figure 6A).

**Table 3:** Current clamp properties of DRG neurons transfected with WT, P1308L, or V1298F Na_V_1.7_R _construct.

Construct	n	R_input_(MΩ)	RMP (mV)	Threshold (pA)
WT	38	605 ± 57	-55.9 ± 1.1	188 ± 14
P1308L	50	749 ± 70	-52.4 ± 1.1	122 ± 10*
V1298F	27	598 ± 46	-55.1 ± 1.5	154 ± 18

* *p *< 0.05 vs WT Na_V_1.7_R _channels.

Previous studies have shown that DRG neurons are able to fire repetitively in response to sustained depolarizing stimuli [29-31]. In this study, 11 out of 38 (29%) small DRG neurons expressing WT Na_V_1.7_R _channels produced 3 or more action potentials, whereas a larger proportion of neurons expressing mutant channels were able to fire 3 or more spikes (64% for P1308L, and 78% for V1298F). Figure 6B shows the mean firing frequency of DRG neurons in response to a series of 1-s current injections ranging from 25 to 500 pA in 25 pA increments, and Figure 6C shows the responses elicited after injecting currents approximately 1.5X and 2X the threshold from the same neurons. Both P1308L and V1298F increased the firing frequency in transfected DRG neurons.

## Discussion

We have identified a new Na_V_1.7 mutation (P1308L) which is only the second IEM mutation to be reported outside domains I and II of Na_V_1.7. The P1308L mutation, from a family with IEM displaying distal extremity pain triggered by warmth, is located within the C-terminus of the channel DIII/S4-S5 linker, and is separated by only 9-10 amino acid residues from three PEPD mutations - V1298F, V1298D, and V1299F - which are located within the N-terminus of this linker. We show here that despite their proximity within the same linker, the P1308L and V1298F mutations have markedly different effects on Na_V_1.7 channel gating properties. P1308L hyperpolarizes activation and slows deactivation, whereas V1298F depolarizes fast-inactivation and enhances repriming. These data provide evidence for a differential role of the N- and C-termini of the Na_V_1.7 DIII/S4-S5 linker in channel activation and fast-inactivation. Our data also show that both mutants produce larger ramp currents and increase DRG neuronal excitability, which provides a cellular basis for pain in patients carrying these IEM and PEPD mutations.

The DIII/S4-S5 linker sequence is highly conserved among sodium channels (Figure 1), suggesting a conserved role in channel function. Crystal structures of a few ion channels, and predicted models of others, depict a voltage-sensor connected to the pore (segments S5 and S6) via the S4-S5 linker [32-37]. The S4-S5 linker potentially interacts with the C-terminal part of S6 which is bent around a glycine "hinge residue" [37], leading to the opening of the channel gate, and proximity of the S4-S5 linker to S6 within each domain has been verified in hERG channels by cysteine substitution [38]. Thus, mutations in S4-S5 linkers are expected to affect activation, consistent with the findings that mutations in these linkers of Na_V_1.7 induce significant hyperpolarizing shifts of channel activation [[17,20,21], and this study].

Several mechanisms may underlie the effect of PEPD mutations within the N-terminal part of the DIII/S4-S5 linker on Na_V_1.7 fast-inactivation: the contribution of residues within DIII- and DIV/S4-S5 linkers to the docking receptor for the fast-inactivation IFMT tetrapeptide [39-42], or predicted mutant-induced perturbation of the α-helical structure of the DIII/S4-S5 linker [25], or this linker potential interaction with the plasma membrane [43]. It is possible that the V1298 contributes to the IFMT docking site, and that V1298F substitution interferes with stable docking of the inactivation particle, impairing fast-inactivation of the mutant channel. There is no direct evidence, however, that N-terminal residues of DIII/S4-S5 linker, including V1298, participate in forming the IFMT docking site. Related to an indirect effect on interaction of the IFMT peptide with its docking receptor is the possible effect of V1298F on the putative interactions of the DIII/S4-S5 linker with the plasma membrane. X-ray crystallographic modeling structure of voltage-gated potassium channel K_V_1.2 places the S4-S5 linker parallel to the membrane inner surface, facing aqueous and lipid environment on opposite sides [43]. By analogy, sodium channel S4-S5 linkers may assume a similar topology, with V1298 in the DIII/S4-S5 facing the membrane lipid layer. Thus the partial positive charges that are present on the hydrogen atoms of aromatic residues [44] may perturb this arrangement in the case of the Na_V_1.7/V1298F mutant, destabilizing the receptor for the fast-inactivation gate and impairing Na_V_1.7 fast-inactivation. This view is supported by the impairment of fast-inactivation by another PEPD mutation V1298D in which a charge is introduced at this position [2,25]. Alternatively, the V1298F substitution may produce an allosteric effect that alters the S4-S5 linker α-helical structure, destabilizing IFMT-receptor interaction, thus impairing fast-inactivation [25].

The important roles of V1298 and P1308, located at opposite ends of the DIII/S4-S5 linker, in regulating channel gating are further supported by identification of disease-causing mutations of corresponding residues in other channels: Na_V_1.1/V1335M (corresponding to Na_V_1.7/V1298), Na_V_1.4/P1158S (corresponding to Na_V_1.7/P1308), and Na_V_1.5/P1332L (corresponding to Na_V_1.7/P1308). Na_V_1.1/V1335M mutation in patients with severe myoclonic epilepsy of infancy replaces the hydrophobic valine residue with the polar methionine, but the effects of this mutation on channel gating has not been studied by patch-clamp [45]. Interestingly, Na_V_1.4/P1158S and Na_V_1.5/P1332L produce hyperpolarizing shift of activation as does Na_V_1.7/P1308L [46-48] and a larger R_resist _of slow-inactivation, but note that Na_V_1.4/P1158S shows a depolarized shift of V_1/2,slow_; slow-inactivation of Na_V_1.5/P1332L was not tested. However, unlike Na_V_1.7/P1308L, Na_V_1.5/P1332L hyperpolarizes steady-state fast-inactivation of the mutant channels [46]. Taken together, these data suggest an important role for a proline amino acid within the C-terminus of the DIII/S4-S5 linker in channel activation, although the effects of this residue on inactivation states appear to be isoform-dependent.

Expression of either P1308L or V1298F induced hyperexcitability of DRG neurons, compared to neurons expressing WT channels. P1308L reduced the current threshold for single action potential (WT: 188 ± 14 pA; P1308L: 122 ± 10 pA), consistent with the role of Na_V_1.7 as a threshold channel [11,12]. While V1298F increased ramp currents, similar to the P1308L channels, it did not cause a statistically-significant reduction in the threshold for single action potentials (WT: 188 ± 14 pA; V1298F: 154 ± 18 pA). However, expression of either P1308L or V1298F increased the number of DRG neurons that fired ≥ 3 spikes in response to a 1-s stimulus (Fig. 6). The ability of both types of mutation to induce DRG neuron hyperexcitability is in agreement with our previous findings of a similar effect of other IEM and PEPD mutations [5,6,18,19,24,26], and provides a cellular basis for pain in patients carrying these mutations.

In summary, the present study shows that the V1298F (PEPD) and P1308L (IEM) mutations, both substituting single amino acids within the DIII/S4-S5 linker of Na_V_1.7 channel, increase DRG neuronal excitability by affecting different biophysical properties of Na_V_1.7. Our results implicate the N- and C-termini of the DIII/S4-S5 linker in different aspects of Na_V_1.7 channel gating, and demonstrate that mutations at those sites differentially affect channel properties.

## Methods

### Exon Screening

Patients were recruited under an approved institutional protocol for research on human subjects. Human variation control DNA panel (25 white males, 25 white females for a total of 100 alleles; Coriell Institute, Camden, NJ) was the source of control samples. All coding exons and flanking intronic sequences, were amplified and sequenced as described previously [18]. Genomic sequences were compared with the reference Na_V_1.7 cDNA [8] using the basic local alignment search tool (BLAST; National Library of Medicine, Bethesda, MD) and Lasergene (DNAStar, Madison, WI). Sequencing was performed at the Howard Hughes Medical Institute/Keck Biotechnology Center at Yale University (New Haven, CT).

### Plasmids

The TTX-R human Na_V_1.7_R _expression plasmid construct generated by Y362S substitution in the cDNA insert described in Klugbauer et al [8] was described previously [28]. The V1298F and P1308L substitutions were introduced into Na_V_1.7_R _using QuickChange XL site-directed mutagenesis (Stratagene, La Jolla, CA).

### Western blot analysis

To examine the expression level of WT or mutant channels, HEK 293 cells were transiently transfected WT or mutant construct together with human β_1_- and β_2_-subunits. After 24-hr incubation, cells were washed once with PBS and lysated with sample buffer. Cell lysates were incubated at 37°C for 25 min followed by brief sonication on ice. Supernatants (10 μl) of cell lysates were loaded on a 4-12% Bis-Tris Gel (Invitrogen) for electrophoresis and transferred onto a Nitrocellulose membrane (30 V for 2 hr). The membrane was blocked with 10% dry milk in TBS-tween (0.1%) overnight at 4°C. After washing, the membrane was cut in half and each half (upper, wells side) and lower (bottom side) were incubated with appropriate primary (room temperature for 2 hr) and secondary antibodies (room temperature for 1 hr). The primary antibody for the upper portion of the membrane was mouse anti-pan sodium channel (Sigma; 1:1000), and for the lower half was the rabbit anti-β-actin (Abcam; 1:5000). The secondary antibodies were polyclonal goat anti-mouse HRP (1:10,000) and goat anti-rabbit HRP (1:10,000), respectively. The blots were washed six times with TBS-tween 20 buffer. The luminescence was induced by Western Lightning Chemiluminescent Reagent (Perkin Elmer Life Sciences, Boston, MA) and detected by film exposure (BioMax XAR, Kodak, Rochester, NY). Band intensities were measured using KODAK MI™ software. The intensities of WT, P1308L, V1298F channels were normalized with the intensities of β-actin of the corresponding lanes to eliminate sample loading variability. The normalized intensity of WT channels was set as 100%, and the level of mutant channels was expressed as the percentage of that of WT channels. Non-transfected HEK 293 cells were used as negative control for antibody specificity.

### Voltage-clamp recordings

Whole-cell voltage-clamp recordings of HEK293 cells expressing wild type (WT), V1298F, or P1308L mutant Na_V_1.7_R _channels were obtained with an Axopatch 200B amplifier (Axon Instruments, Foster City, CA). All experiments were conducted at room temperature (20-22°C). Fire-polished electrodes (0.6-1.3 MΩ) were fabricated from 1.6 mm outer diameter borosilicate glass micropipettes (World Precision Instruments, Sarasota, FL). The pipette potential was adjusted to zero before seal formation, and liquid junction potential was not corrected. Capacity transients were cancelled and voltage errors were minimized with 80-90% series resistance compensation. Currents were acquired with Clampex 9.2, 6 min after establishing whole-cell configuration, sampled at a rate of 50 or 100 kHz, and filtered at 5 kHz.

For characterizing channel biophysical properties, HEK293 cells stably expressing either WT or mutant (V1298F or P1308L) Na_V_1.7_R _channels were generated as described previously [21]. For current-voltage relationships, cells were held at -100 mV and stepped to a range of potentials (-80 to +60 mV in 5 mV increments) for 100 ms. Peak inward currents (I) were plotted as a function of depolarization potential to generate I-V curves. Activation curves were obtained by converting I to conductance (G) at each voltage (V) using the equation *G = I/(V-V*_*rev*_), where V_rev _is the reversal potential that was determined for each cell individually. Activation curves were then fit with Boltzmann functions in the form of *G *= *G*_*max*_*/{1+exp [(V*_*1/2, act*_*-V)/k]}*, where *G*_max _is the maximal sodium conductance, *V*_*1/2,act *_is the potential at which activation is half-maximal, *V *is the test potential, and *k *is the slope factor.

Steady-state fast-inactivation was achieved with a series of 500 ms prepulses (-140 to -10 mV in 10 mV increments) and the remaining non-inactivated channels were activated by a 40 ms step depolarization to -10 mV. Steady-state slow-inactivation was determined with 30 s prepulses at voltages ranging from -130 to +10 mV followed by a 100 ms hyperpolarization at -120 mV to remove fast-inactivation. Remaining available channels were activated by a 20 ms test pulse to -10 mV. Peak inward currents obtained from steady-state fast-inactivation and slow-inactivation protocols were normalized with the maximal peak current (I_max_) and fit with Boltzman functions:

where *A *represents the offset, *V *represents the inactivating prepulse potential, and *V*_1/2 _represents the midpoint of the inactivation curve. In the text, *V*_*1/2,fast*_*and V_1/2,slow _*were used to represent the midpoints for steady-state fast-inactivation and slow-inactivation, respectively.

Deactivation was examined using a short (0.5 ms) depolarizing pulse to -20 mV followed by a 50 ms repolarizing pulse to potentials ranging from -100 to -40 mV in 5 mV increments. Deactivation kinetics were calculated by fitting the decaying currents with a single exponential function. Ramp currents were elicited with a slow depolarizing voltage ramp from -100 to +20 mV at a rate of 0.2 mV/ms. The absolute ramp current amplitude was normalized to the maximal peak current obtained by the I-V protocol.

The recovery of Na_V_1.7_R _channels from fast-inactivation (repriming) was examined using a two-pulse protocol with the interpulse intervals varying from 2 ms to 300 ms. Repriming was studied at four recovery potentials (-100, -90, -80, and -70 mV) for all three channels, and V1298F was also tested at an additional recovery potential of -60 mV. Recovery rates were measured by normalizing the peak current elicited by the test pulse with that of prepulse at 0 mV after various recovery durations (2-300 ms) at different recovery potentials, and plotted as a function of recovery potentials. Recovery time constants were calculated using mono-exponential fits of the recovery rates at different recovery duration.

The pipette solution contained (in mM): 140 CsF, 10 NaCl, 1 EGTA, 10 dextrose, and 10 HEPES, pH 7.32 (adjusted with CsOH), and the osmolarity was adjusted to 308 mOsmol/L with sucrose. The extracellular bath solution for voltage-clamp contained (in mM): 140 NaCl, 3 KCl, 1 MgCl_2_, 1 CaCl_2_, 10 dextrose, 10 HEPES, pH 7.35 (adjusted with NaOH), and the osmolarity was adjusted to 315 mOsmol/L with sucrose. Tetrodotoxin (TTX, 300 nM) was added to the bath solution to block endogenous voltage-gated sodium currents in HEK293 cells, permitting currents from WT or V1298F or P1308L mutant Na_V_1.7_R _channels to be recorded in isolation.

### Isolation and transfection of DRG neurons; current-clamp recordings

DRG neurons were isolated from 1- to 4-day-old Sprague Dawley rats as described previously [49]. WT or mutant Na_V_1.7_R _channels were electroporated into DRG neurons together with GFP constructs (channel:GFP ratio of 5:1) using Rat Neuron Nucleofector Solution (Lonza, Walkersville, MD) as described previously [49]. Small (20-25 μm) DRG neurons with robust green fluorescence were selected for current-clamp recording 18-48 hr post transfection. Whole-cell configuration was obtained in voltage-clamp mode before starting current-clamp recording. Input resistance was determined as the slope of the linear fit of the hyperpolarizing responses to a series of current steps from 5 to 40 pA in 5 pA increments. Current threshold for action potential generation was determined by a series of depolarizing currents in 5 pA increments. Repetitive firing frequency was examined in response to a series of 1-s current steps from 25 to 500 pA in 25 pA increments.

The pipette solution for current-clamp recording contained (in mM): 140 KCl, 0.5 EGTA, 3 Mg-ATP, and 5 HEPES, pH 7.3 (adjusted with KOH), and the osmolarity was adjusted to 308 mOsmol/L with sucrose. The extracellular bath solution for current-clamp recording contained (in mM): 140 NaCl, 3 KCl, 2 MgCl_2_, 2 CaCl_2_, 10 dextrose, 10 HEPES, pH 7.3 (adjusted with NaOH), and the osmolarity was adjusted to 315 mOsmol/L with sucrose.

### Data analysis

Data were analyzed using Clampfit 9.2 (Molecular Devices) and OriginPro 8 (Microcal Software, Northampton, MA), and presented as means ± SE. Unless otherwise noted, we used one-way ANOVA followed by Tukey post hoc test for multi-group analysis for statistical significance. For comparison of firing frequency between neurons transfected with WT and mutant channels was performed with Mann-Whitney test. We report exact p values except when they were smaller than 0.001, which were reported as p < 0.001.

## Abbreviations

IEM: inherited erythromelalgia; PEPD: paroxysmal extreme pain disorder; DRG: dorsal root ganglion; SCG: superior cervical ganglion; TTX: tetrodotoxin; RMP: resting membrane potential; TTX-S: tetrodotoxin-sensitive; TTX-R: tetrodotoxin-resistant.

## Competing interests

The authors declare that they have no competing interests.

## Authors' contributions

XC collected, analyzed and interpreted electrophysiological data. SDD-H participated in the experimental design and interpretation of the data. LT identified the P1308L mutation in genomic DNA, generated the mutant P1398L and V1298F constructs, and established the stable cell lines. DAW and TZF collected and interpreted patients' clinical data and confirmed an IEM diagnosis. SGW conceived the project, participated in the experimental design and interpretation. XC, SDD-H, TZF, and SGW participated in writing of the manuscript. All authors read and approved the final manuscript.
